# The Incredibility of Veracity

**Published:** 1906-01

**Authors:** 


### TEXAS MEDICAL JOURNAL.

AUSTIN, TEXAS.

A MONTHLY JOURNAL OF MEDICINE AND SURGERY.

EDITED AND PUBLISHED BY
F. E. DANIEL, M. D.
Mrs. F. E. DANIEL, Business Manager.

Published Monthly at Austin, Texas. Subscription price $1.00 a year in advance

### THE INCREDIBILITY OF VERACITY.

A MORNING CALL; THE PROFESSOR DROPS IN.

I had an interesting experience yesterday. I was sitting in my office looking over my bills, payable and receivable, figuring how I was going to make known expenses come within the range of possible receipts, when the door opened softly and there glided in a tall, sallow, long-haired individual, with eyes deep and dark; "eyes like Mars, to threaten and command." I was scared. He said:

"I am Professor Schlopjahr of the University of Texas, and I want to ask you a question."

"Fire when you are ready, Gridley," I said, softly grasping a paper-cutter, and looking around for something to throw, if necessary.

"Why should the spirit of mortal be proud?"

"I give it up," said I. "Search me."

Seeing that he was loaded, I winked at Miss Sally, my pretty stenographer, and she got busy.

"Take a seat," said I.

"I'll tell you, then," he said. "It's man's vanity. He is the

---

In "The Thousand-and-Second Tale of Scheherazade" (Poe) the eighth voyage of Sinbad the Princess tells the Caliph that Sinbad saw railroads, telegraphs, balloons, photographs and other wonders and described them, until his credulity revolted. "That's the limit, Schezzy. Now if you had told me about that blue cow that stands on the back of a turtle and holds up the earth on her four hundred horns, I might have believed that, for I have seen something like that in the Koran. The bow string for you, Schezzy, my dear," says he, or words to that effect.

vainest thing in existence unless it is the goose that stoops to enter a barn door for fear he will bump his head. It is his vanity that prompts him to believe and say that he is the biggest thing in all the Universe, the chief end of God's purpose, and the one object of His solicitous care and providence; that he, a microbe, can offend God; can propitiate Him,—by prayer and fasting—fasting! Oh, the absurdity of the thing!"

The professor paused and wiped his brow with a red handkerchief and continuing, said:

"From his own standpoint he is a big thing. The reason is, that he looks at himself from too close range, through a magnifying glass, as it were. It seems never to have occurred to him to look at himself at long range, and in relation to other things. His 'Universe' is what he sees; what comes within the range of his five little senses. He can appreciate nothing which is beyond his consciousness; and I may say the same of all animal organisms, even to the lowest of those who inhabit the waters of the earth, the little fellows whose organisms consist of a few of those wonderful things we call 'cells,' batteries, store houses of energy and protoplasm, 'live' matter. Say the *rhizopods,* for instance; those industrious little fellows who build the coral reefs, whose product in early ages formed the mummulite, of which the pyramids are partly constructed. They doubtless conceive the Universe to be the element in which they live, bounded only by the range of their senses. So the ant, the bee, the ephemera, even, whose life consists of a fraction of time, measured by man's idea of time, is a few hours; yet in that time, which to them is relatively as long as man's life, they live, fulfill their missions, reproduce their kind, and generation after generation follows, as with men, in quick succession. From their standpoint, man is an inconceivably big thing, something far beyond the range of their senses and their comprehension; as much so as bigger things, the Universe, are beyond the comprehension of man. Man thinks that this little earth we are on is the world. He looks into the heavens and sees the suns of other systems with their worlds whirling around them, doubtless inhabited by living organisms similar to those on earth, perhaps far more highly organized and developed, because some of those worlds are older; he realizes they are worlds, and acknowledges, perhaps, that they may be inhabited, yet clings to the old fable of creation and of Adam and Eve, and of the flood and the ark, and other myths formulated in the ages when nothing was known of astronomy as now understood, or geology or any other science. He still thinks that the heavens and all their glories, the stars, moons and planets, are a secondary consideration to man, all made for his especial benefit; mere appendages, as it were, of this great world, this little eight-thousand-mile ball which floats so inconspicuous in the ocean of space."

The professor looked at me as if he expected me to butt in, but I kept mum. He started fresh:

"It is astonishing that in this enlightened age men cling with such pertinacity to the traditional fiction which originated with a barbarous, nomadic people, the Jews, some four thousand years ago, that God selected this little planet, the earth, a mere speck of matter in the vast space of the Universe, out of all His countless millions of worlds, and visited it in person, talking to and guiding, making promises to, and 'covenants' with and directing, and issuing 'commandments' to that particular little handful, that species of microbe, the Jews, who call themselves His 'chosen people'; putting a rainbow in the sky as a 'sign' that he was still in a good humor and intended to keep His word! Men cling to it in spite of reason; a traditional fiction, originating in the active imagination of an ignorant, superstitious and highly imaginative race of Orientals, and imposed by Moses doubtless for a purpose,—for the purpose of making the Jews obey his laws. I think that Moses knew better. I think he issued 'commandments,' made laws in the name of God, to make the people afraid to disobey.

"Rot," said I. "Don't you know that Jonah swallowed the whale all right,—for sure? (The professor glared at me, but continued:)

"Yes, from his own standpoint, man is the whole thing. From the standpoint of the inhabitants of smaller worlds, microscopic to us, say, those of Phobos and Deimos, the tiny satellites of Mars, man is inconceivably large, no doubt; something beyond the power of their little senses to comprehend. Agnes Giberne (*"Story of the Sun, Moon and Stars"*) thinks those little worlds may be peopled. Phobos is 7.45/100 miles in diameter, and Deimos 6.2/10, about one-twelve-hundredth part of the earth's little 8000-mile diameter. In speculating on the presumed inhabitants of those tiny worlds she says:

"The vanity of men being generally in the direct ratio of their mediocrity, the microscopical reasoning mites which doubtless swarm upon their surface, have also, perhaps, permanent armies which mutilate each other for the possession of a grain of sand."

"She might have added that these reasoning mites, also, doubtless have a superstition, based upon some ancient fable of hoary antiquity, that they call a religion, and in times of danger they doubtless supplicate something to save them, to 'hold them in the hollow of its hand,'—even as men on earth do."

I said "Fudge."

"Let us look at the earth, now," said the the professor, "and its inhabitants at a little distance,—a little longer range,—from a more remote standpoint. Let us look at it, say, from our big brother planet, Jupiter. He is only 460,000,000 of our little miles away.

"We will suppose that Jupiter has long since cooled down to 'a point between boiling and freezing,' when water, the parent of all life, animal and vegetable, is possible; and that countless ages ago life made its appearance on its crust, as it did on that of the earth, and developed through countless forms to a stage corresponding, we will say, to man. That is to say: If the same conditions exist, or have existed, on Jupiter that developed man on earth from the protozoa, the *verma* and other invertebrates, up to and through the fishes and reptiles and other vertebrates; up to and through the quadrupeds, then the inhabitants of Jupiter are, or rather were once, men. There is no data upon which to found even a conjecture as to the form and shape of the highest form of animal life on that big planet. As I have said, Jupiter is older than the earth. Supposing it to once have been in a condition favorable to the origin of life (which I believe to be chemical, the action of oxygen upon the nitrogenous compounds and other salts in the water), evolution from the lowest form of animal life, the protozoa, has been going on much longer than on the earth; and supposing that it took precisely the same course on Jupiter that it took on earth, I say, the inhabitants have long since passed the man stage. If the earth lasts long enough, man will continue to evolve until he will reach the form and shape of *something*, there is no guessing what, that will be as unlike man as man is unlike any of his ancestors, the fishes, for instance. There will then be, I doubt not, individuals amongst these beings, who will utterly scout and repudiate their descent from such an insignificant thing as *man*, as there are now amongst men, individuals, wise ones, who indignantly deny their undoubted descent, not only from the worm, but from the anthropoid apes, some of whom are superior today to some individuals of the human species. Take Darwin's old male gorilla, for instance, who turned and faced his pursuers, alone, to give time for the females and the young of the tribe to escape; and compare him with certain races of *men*, the anthropophagi, some of whom eat their babies. Man will evolve into something, in the course of untold ages, totally unlike man. What it will be, there is no way of knowing. He may retain his shape, in part, at least, retaining only the useful parts and exaggerating them by development from constant use, as the arms, the hands, discarding those that are useless, as the toes. The toes are bound to go. They perform no function. Shut up in a shoe they are useless, and in time will be eliminated. Already in certain individuals the little toe is rudimentary. In the ape stage of man they were useful, were indispensable; they were used in climbing. The seal, the sea lion and the walrus represent the transitional stage from fish to quadruped. They must adapt themselves to environment. They have to climb out of the water on rocks and icebergs, and in time their flippers will become feet with claws, as we see in the turtle and in the otter and the beaver. They may develop legs and arms, and bye and bye, perhaps, hands and feet; who knows? It all depends upon environment. Man may become larger, retaining his most useful members, like the horse of today, which is descended from a little quadruped about the size of a fox, and having five toes; or he may become much smaller, like the little gray lizard that runs on the fence, whose ancestors were the prehistoric (Miocene) *Dinosaurs,* the 'terrible lizard,' sixty feet long and weighing several tons. It all depends on environment. Animal and plant life must adapt itself to environment or perish.

"Man's power of adapting himself to his surroundings is one of his highest characteristics. Why has the giraffe a long neck, and, like the cow, the camel and the horse, prehensile lips? Environment; the struggle for existence developed it. In early ages something which is now 'giraffe,' was so situated that it had to eat the leaves and buds of trees, or starve. The neck was constantly elongated to enable him to do so. It is well known that mustang ponies, used for countless generations to grazing on the prairies, can hardly lift their heads higher than a level with the back, and when taken to hill country always stumble in going down hill. Were men, or any race of man, so circumstanced that the arms and the hands could not be used throughout many generations, and it became necessary to seize their food with their lips, as do cattle, they would develop the prehensile lips; and if, like the giraffe, they had to reach up for their food, they would develop giraffe neck. There is no doubt about that."

I said "Oh, Tommy rot!"

"Nevertheless, we being still men," said my visitor, "we will suppose, and for the sake of argument, assume, that the inhabitants of Jupiter (we are assuming that there are inhabitants on Jupiter), are still men. We don't know what they are nor what men on earth will be by or before the time when the earth, having become like our moon, a frozen, lifeless world, will slacken its speed and drop into the sun and burn like a feather in a hot stove; or Jupiter's pull on it, having overcome the centrifugal force which carried it around the sun so long as the density of the earth enabled it to maintain an equilibrium, will elongate more and more the ellipsis of the earth's orbit and will attract her to himself. There will then be a collision, a crash of worlds, and the earth will fly into fragments, such as we encounter in aerolites; the debris of extinct worlds; will become 'cosmic matter,' and its particles, coming in reach of some center of attraction of new world formation, will enter into new combinations, and form part of some future great sun. This sun in time will condense, as all suns do, form an equatorial belt of burning gas or fluid which, in time, will break up into spherical bodies. They will become its planets, and begin anew the ceaseless round of birth, growth, decline, decay and death of worlds which is going on, and forever has gone on in the incomprehensible scheme of the universe."

I said: "There'll be something doin', then, sho' nuff."

"We then will call the Jupiterians men. Man can not conceive of anything higher than himself as occupying a world," said he.

"Jupiter is twelve hundred times larger than the earth. Let us suppose that its inhabitants are correspondingly larger; that they are twelve hundred times as large as we; that their senses are correspondingly more numerous and more acute than earth-man's, and that, therefore, instead of five senses—earth-man's—the Jupiter man has twelve hundred times five, or about six thousand senses, all twelve hundred times more acute, more highly developed than man's. *His* universe will be limited by the range of his senses. Hence, he can take, as it were, a broader view, and have a grander conception of the universe than we. He can grasp and comprehend things which are a long way out of our reach; and still there will be much, the most,—the greater part of that thing we speak of so vaguely as the 'universe,'—beyond his comprehension. He will have to say, as we do: 'There is no limit to the universe.' Jupiterian mind can not conceive of a beginning or a creation; for, to do so, we must contemplate a time when there was nothing, which is unthinkable. Nor can there be a limit to space, distance, or time. Jupiterian mind can not grasp the idea of eternity. Measured by our conceptions of time, it is inconceivably great.

"Scientific men say that our nearest neighbor in the great stream of energy and matter which we call the Milky Way, the procession of suns and systems of worlds,—solar systems,—is Alpha, in the Centaur, and that it is twenty-five trillions of miles from our sun. Sirius is fifty-eight trillions of miles off. That means just nothing at all; it conveys no idea. Yet, were it possible for one to go to Alpha or Sirius, he would find the next nearest neighbor equally remote. Yet, who knows but that if it were possible to go far enough through space, away beyond the remotest sun or system revealed to man by his ingenuity, far away, inconceivably far, farther than a ray of light, traveling one hundred and eighty-five thousand miles in a second, could penetrate in a million years, there may not be, beyond the limits which our little minds put to space, matter of a very different composition to suns, planets, comets, 'ether,' or anything we know or conceive of, and which bears the same relation to what we see and know, our environment, that land,—say the east coast of Asia, or the west coast of America, bears to the Pacific Ocean, the 'Universe,' the environment of millions of living things; of the *rhizopod* and the *foraminifera,* whose shells built the Cliffs of Albion, when what is now England was the bottom of the ocean; the *caelenteria,*—all the protozoa, in fact?

"It is not a difficult thing to imagine," continued the professor, "that these minute living things, living in great communities as they do, each has his individuality, his 'ego,' his own affairs, his hopes and fears. Perhaps he has a 'religion,' which shall express his conception of a Deity, and when the winds and the waves come and knock over his little house (the coral builders, for instance), he will implore to be 'spared from the wrath of an offended God.'

"I have no doubt that each of these microscopic things regards himself from his standpoint as we regard ourselves from our standpoint,—as a *big thing;* of vast importance in the Universe! I can imagine one of the most 'advanced' of these microbes, a *moneron,* or an amoeba, for instance (and doubtless they have 'scientists' amongst them), arguing the question of time and eternity, of the limits of the Universe, even as *we* do. Their Universe is, like ours, boundless. It is the Pacific Ocean. They can no more comprehend the fact that if they could *go far enough* they would come upon land, than we can comprehend the possibility of there being some other form of matter beyond the possible range of our poor little senses. The 'scientists' would stoutly contend that the 'Universe,' *their Universe*—the Pacific Ocean— always *was* and always *would be.* But let us go back to Jupiter:

"We will suppose now, the most 'advanced' scientist on Jupiter, a professor of astronomy—a Simon Newcomb, a Barnard, or a Proctor—in the Jupiterian University to be lecturing on astronomy, his subject being 'The Earth.' Remember, he and his class have six thousand senses, each one thousand two hundred times sharper than ours; and everything, their telescopes and microscopes, are on the same relative scale. He will say:

" 'Gentlemen, the minute point of light, the tiny speck of matter (a mere mote in a sunbeam), which we call the earth, is now visible. I turned our great reflector on it, and will presently show you a fifty-Jupiterian-foot image of it on the screen. You can examine it with the naked eye and easily make out the continents and the oceans. It is about one-twelve hundredths of a mile in diameter, the smallest heavenly body known to us.

" 'But what will interest you more is the fact that it is inhabited; that is, there are crawling on its surface what appear to be living things. They are rod-shaped, and should, therefore, be called 'bacteria,' but for the fact that *bacteria* are vegetable cells, and science has reason to believe that these are animals. They are bifurcated; that is, they are two-legged, like exceedingly diminutive Jupiterians, and appear to consist essentially of a body, two inferior and two superior 'processes,' not unlike arms and legs, and a spherical development at the upper extremity, not unlike a head, and which sphere we compare to the nucleus of the nucleated 'cell,' and infer that it is the center of the energy which actuates the organism, giving it power of motion, and we must infer, sensation. Look! The little speck, the earth, is swarming with them. Now they are in great commotion. Something has happened. They are fighting among themselves for possession of part of the earth's surface, or some 'great' cataclysm, such as we experience here sometimes, an earthquake or a storm perhaps, but on a scale to us, microscopic, has taken place, and behold, they fall on their knees as we do, and implore their conception of a wrathful deity to save them from the destructive power of the physical forces of nature, which control and operate even their little speck of matter, the earth, their 'world,' as they do great Jupiter and our elder brothers, Saturn, Uranus, and Neptune. But, it seems that the 'good' and the 'bad' perish, all the same, and alike, even as they do here on great Jupiter, despite priest and prayer.

" 'Now, gentlemen,' continues the professor, 'I attached the new patent tele-photo-microscope to the reflector, thus magnifying the microbes 50,000 diameters, and have made a series of instantaneous photographs in quick succession of that part of the earth which is turned towards us and on which so much commotion amongst the mites is taking place; I have placed them in our great revolving kinetoscope and will now project them upon the canvas that you may see the 'inhabitants' in motion, and study them at your leisure. I will lecture tomorrow upon these interesting micro-organisms, the 'reasoning mites' of the earth.'

"It would be interesting to know what it was that the professor saw; whether it was the earthquake at Lisbon, a West Indian cyclone, the eruption of Pelee, or the Russo-Jap war in Manchuria."

I said: "Professor, you are cynical and pessimistic."

"Pessimistic? No, I'm not pessimistic. I take a broader and better view. I sometimes think that 'the poor Indian whose untutored mind sees God in every breeze' was 'broader,' less 'pessimistic,' and nearer right, 'untutored' though he be (*and for that very reason*), than the tutored doctrinarian who conceives God to be in *his* image,—*anthropomorphic,* man-shaped, a talking God, with human voice and speech, speaking Hebrew.

"How do we know, in fact, that the Universe,—I mean in its immensity, its totality, and not our narrow knowledge of it, that immensity which transcends finite conception, is not, itself, a living organism? Was not Pope right when he said:

> " 'All are but parts of one stupendous whole,
> Whose body Nature is, and God the soul'?

"Suppose a microscopic parasite, embedded in the skin of an elephant, an *acarus,* an itch insect, should have intelligence and senses which enable him to look at himself from his own standpoint as man looks at himself from his standpoint; to have an appreciation of his environment, and realize that his 'Universe' is his conscious apprehension of his surroundings, his immediate vicinage, elephant skin and hair-forests. Do you suppose that he could understand the movements of the elephant or the sounds made by him, or estimate his size, or begin to grasp the idea that it is a living thing, identical in nature with its own little life, and all other animal life, differing only in degree, in development and complexity? Could he understand the elephant, even physically, any more than man can understand that which is beyond the range of his consciousness, his senses, the totality of the Universe?

"You see," continued the professor, "there is no 'little' or 'big.' Everything is relative. What is a 'cell'? We say it is a speck of protoplasm, with a nucleus, its center of 'energy,' endowed with sensation and motion, and an intelligence or understanding, shall I say,—a soul that guides it unerringly to the fulfillment of its life work. A 'cell,' from which all organisms, large and small, animal and vegetable, spring, and of which all tissues are constructed. That the nucleus is the seat of the potential energy can not be questioned. In some cells it has been shown, too, that smaller bodies called 'granules' are found within the protoplasm, smaller than the nucleus, and apparently of different composition. These bodies have been thought to be 'nutritive,' and that they contribute to the building up of tissue in some way.

"Let us take a sun, for instance, the center of a solar system, and liken the whole to a cell. Let it represent the nucleus. Take the planets which revolve around it. Let them represent the smaller or nutritive bodies (granules). Take the inter-space, which is hypothetically occupied by *ether*. Let that space represent the protoplasm, the body and substance of the cell. How do we know that each solar system is not a living cell—*writ large*—and that the Milky Way may not be an exaggerated lymph channel, carrying all the millions of suns and systems to the predestined (or selected) point where they may perform their function in the economy of some inconceivably great, Living Thing, repairing waste, building up,—as we know suns and systems are continually doing, —perishing, discharging their energy, exhausting themselves, and being re-organized and rebuilt?

"Indeed, Lowell (in 'The Cathedral') hints at some such thought when he says:

> " 'In the circulation, swift, of Deity,
> Suns and systems inconspicuous float,
> Like tiny blood-discs in our poor mortal veins.' "

Here the professor glared at me.

"Speaking of blood," he said, rising and coming toward me, "I have an idea."

He was much excited, and the muscles of his face twitched, and his long, bony hands worked convulsively, while "his eyes had all the seeming of a demon's that is dreaming," and I was scared for sure.

Just then, at this interesting moment, the door was thrown open, and Dr. Worsham and two attendants from the asylum entered, and seizing the professor, one said, "Here, Johnson, come out of this; this won't do, you know," and they hustled Professor Schlopjahr back to "The House of the Foolish."

